# Hybrid Prosthesis versus Overdenture: Effect of BioHPP Prosthetic Design Rehabilitating Edentulous Mandible

**DOI:** 10.1155/2023/4108679

**Published:** 2023-06-29

**Authors:** Hanan Mohsen Al-Asad, Mahmoud Hassan El Afandy, Hebatallah Tarek Mohamed, Magda Hassan Mohamed

**Affiliations:** ^1^Faculty of Dentistry, Aden University, Aden, Yemen; ^2^University of Science and Technology, Aden, Yemen; ^3^Faculty of Dentistry, Ain Shams University, Cairo, Egypt

## Abstract

**Aim:**

To compare the BioHPP (biocompatible high-performance polymer) as a substructure for the hybrid prosthesis versus the BioHPP bar supporting and retaining implant overdenture by radiographic evaluation to identify bone height alteration around the implants and to evaluate satisfaction based on visual analoge scale questionnaire.

**Materials and Methods:**

Ill-fitting mandibular dentures were chosen for 14 fully edentulous male patients with adequate dental hygiene, enough interarch space, and free of systemic diseases and parafunctional habits. Patients who received new dentures (CDs) were randomly allocated into each group using computer software, and four interforaminal implants were inserted in parallel using a surgical guide. Three months after osseointegration, the patients received either CAD–CAM BioHPP framework hybrid prosthesis (Group I) or BioHPP bar supported and retained overdenture (Group II). Using digital preapical radiography, the bone loss is evaluated 6, 12, and 18 months after insertion. The subjective patient evaluation was done using a questionnaire based on the VAS includes five points for chewing, comfort, esthetics, speech, oral hygiene, and general satisfaction.

**Results:**

The overall marginal bone loss (MBL) revealed that Group I (hybrid prosthesis) was more than Group II (bar overdenture) at all intervals in the anterior and posterior implants' mesial and distal surfaces. The patient satisfaction survey results showed that, after 18 months, the difference was statistically not significant between them all (*P* > 0.05) except for the comfort (for the overdenture group, 4.43 ± 0.53 while the fixed hybrid was 5.00 ± 0.00).

**Conclusion:**

BioHPP framework material is an alternative material for implant rehabilitation of edentulous mandible with minimal MBL in BioHPP bar overdenture compared to BioHPP hybrid prosthesis.

## 1. Introduction

The most difficult aspect of oral rehabilitation is restoring lost structures with proper function and esthetics. Implants dentistry has increased the quality of prosthodontic rehabilitation. Dental implants have a high success rate in patients with bad denture experiences [1]. Treatment options include fixed and removable prosthesis in many forms and using various types of implants and attachments. Compared to the conventional fixed prosthesis, implant-supported overdentures, and hybrid prostheses support the face [2, 3].

The implant-supported hybrid prosthesis is considered a proper line of treatment if porcelain-fused metal fixed restoration does not satisfy a patient's demands for esthetics, phonetics, oral hygiene, and oral comfort [4]. It is indicated that when the bone quantity and quality are sufficient to install the required number of implants (usually four or more) [5]. This prosthesis reduces the desired impact force in functional occlusion [6]. It is also cheaper and has a satisfactory esthetic [7]. Implant success needs accurate loading, where Brånemark [8] stated that the prosthesis precision fit should be at a 10 *µ*m level.

Implant overdentures are usually supported by bars and other rigid attachments connected to multiple implants. Implant overdenture bars are traditionally fabricated by casting methods, which are time-consuming and need many laboratory steps. Recently, the bar framework can be made by CAD/CAM technology [9, 10]. CAD/CAM technology has enabled high-quality dental restorations in less time, improving efficacy, and providing new materials for dental prostheses [2, 11, 12]. Moreover, prosthesis manufactured by the CAD/CAM process gives outstanding clinical success [13, 14].

Polyether ether ketone (PEEK) has recently attracted more attention for making superstructures on dental implants [15]. It is an organic thermoplastic polymer with perfect mechanical and esthetic properties that is stable at high temperatures and can be used as a replacement [16]. It has a modulus of elasticity compared to the bone, resulting in better stress distribution to the surrounding tissues and a slower bone resorption rate [17]. In addition, its properties can be modified to be compatible with the biological consideration by adding other materials such as carbon fibers (carbon fiber reinforced/CFR-PEEK) and ceramic microparticle fillers (biocompatible high-performance polymer; BioHPP) [18].

The BioHPP is based on the PEEK polymer and is used in prosthodontics. This material has good physical properties, high-temperature stability, and resistance to chemical damage. The unique ceramic filler (with a grain size of 0.3–0.5 m) optimizes the mechanical properties; the tiny grain size also gives homogeneity [19, 20]. It is biocompatible, bioinert, and radiolucent, and it is compatible with carbon and glass fibers [21, 22] CAD–CAM high-performance polymers (HPPs) are alternative materials to titanium and zirconia. BioHPPs have been widely used as a framework material for fixed and removable prostheses and implant-supported prostheses, resulting in restorations with superior and more repeatable mechanical properties [23, 24].

Considering that bone-anchored prosthesis is designed to survive a lifetime in the oral environment, a pathologic decline in bone level could result in the loss of bone anchorage of the implant, so it is critical to understand what causes bone resorption. There is controversy over several factors that affect implant success, particularly marginal bone loss (MBL) [25]. Therefore, this research aimed to compare the BioHPP used as the substructure for the hybrid (implant-fixed detachable) prosthesis to the BioHPP bar supporting and retaining implant overdentures by measuring the marginal bone height alteration around the implants through radiographic evaluation and assessing patient satisfaction through the use of a visual analog scale. The null hypothesis of this research was that the crestal bone loss and patient satisfaction are comparable in the two-treatment plan; BioHPP hybrid prosthesis and BioHPP bar supporting and retaining implant mandibular overdenture.

## 2. Materials and Methods

### 2.1. Patient Selection and Study Design

This research was listed as a randomized controlled clinical trial and was reviewed and approved by the research ethics committee (FDASU-RecD/R041808) of the Faculty of Dentistry, Ain Shams University, following CONSORT guidelines for the clinical trial registration (*NCT05468983*). Fourteen fully edentulous patients from the Department of Prosthodontics Outpatient Clinic, Faculty of Dentistry, Ain Shams University, were selected. The patients potentially eligible for this study were identified, and the researcher contacted them to explain the research and ascertain their interests. Written informed consent from the patients was obtained after explaining the study protocol to them.

#### 2.1.1. *Inclusion and Exclusion Criteria* [21]

Male patients ranging in age from 50 to 65, sufficient bone quantity and quality in the mandibular interforaminal region to install standard implants of at least 10 mm length and 4.2 mm diameter, good oral hygiene, and normal maxillo–mandibular relationship, on the other hand, exclusion criteria included the following: patients who have evidence of systemic diseases or disorders, smokers, parafunctional habits, or previous chemotherapy or radiotherapy treatment.

### 2.2. Surgical and Prosthetic Procedures

The general, extraoral, and intraoral exams were performed to determine that the conditions for the intended implant treatment were suitable. All patients received dentures with a balanced occlusion and modified acrylic teeth. The participants were asked to wear the dentures for 2 months before implant insertion. They were divided into two groups by random allocation software (an Excel sheet) blinded by an independent person not involved in the study protocol. All patients were rehabilitated with four parallel interforaminal implants and the BioHPP framework for Group I (BioHPP fixed hybrid prosthesis). A BioHPP bar supported and retained overdenture Group II (BioHPP bar overdenture).

#### 2.2.1. Patient Imaging and Case Planning

**Figure 1:**
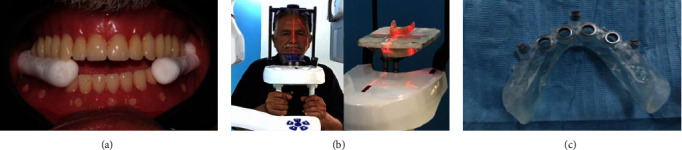
(a) A patient biting on centric occluding relation, (b) dual scan technique, and (c) surgical guide.

A virtual implant placement plan that considers the implant's size, length, diameter, and angulation was made using cone-beam CT software. The composite markers' polished lingual, labial, and buccal surfaces were placed on the mandibular denture already in place, which was then used as a radiography template. The dual-scan method (1. VGI, QR, Verona, Italy) used CBCT. Each patient has a first scan done while chewing bilaterally on cotton rolls in centric occlusion and a second scan done with just the denture in place. The virtual model planning program specifies the anchor pins for the surgical guide and the locations for implant insertion. The development of a mucosal-supported stereolithographic surgical template with four sleeves placed over corresponding implant locations (form lab) as illustrated in Figure 1(a), 1(b), and 1(c).

The use of “chlorohexidine” mouthwash (Oraldene, Hikma Pharma, Egypt) was advised to patients. The surgical guide was disinfected in a “2% glutaraldehyde” solution for 15 min. All surgical equipments were sterilized before use. The patient has received anesthesia for bilateral mandibular nerve blocks. (articaine chlorohydrate 1 : 1,00,000 with adrenaline), local infiltration, and anesthesia were then administered to the operative area to lessen bleeding. The computer-guided stent was inserted over the mandible using the centric occluding relation (COR).

**Figure 2:**
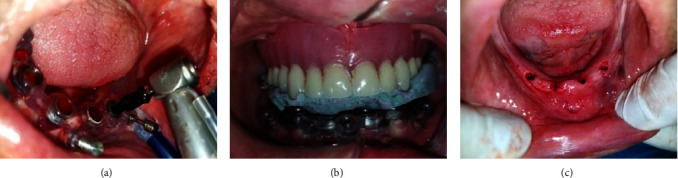
(a) Surgical guide fixation, (b) sequence drilling and (c) final position of implants.

The universal surgical instruments (the JD Italian Guide) provided by the creator of the real guide were used for the osteotomy preparation. As per factory specifications, osteotomy sites for the implants were systematically prepared using a series of drills until completion. The identical oral and maxillofacial surgeon used a nonsubmerged flapless surgical approach to insert the four implants (Trate AG, Switzerland; Roott, two-piece dental implant) in the interforaminal region of the mandible, as illustrated in Figure 2(a), 2(b) and 2(c).

### 2.3. Prosthetic Procedures

**Figure 3:**
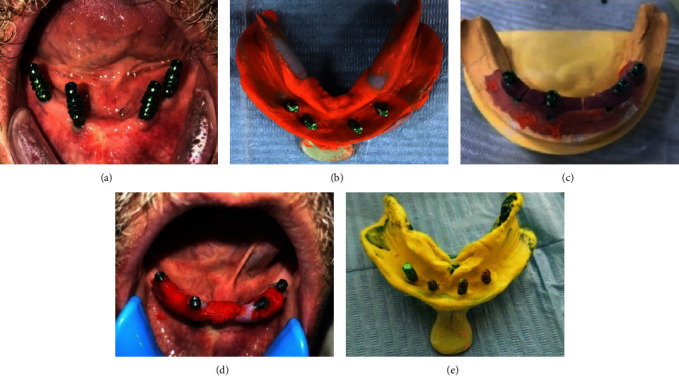
(a) Implant impression copings, (b) implant analoges attached to the impression copings, (c) verification jig section, (d) intraoral splinting the jig, and (e) final impression and reattached anlage to the final impression copings.

The cover screws were removed 3 months after the first procedure, and healing abutments were fitted in their place. The extended transfer impression copings were threaded onto the implants and the open tray impression technique was performed. After removing the set material, the unique tray was filled with rubber-based impression material (Zhermack, Italy, Elite HD + a-silicone impression material) and put into the patient's mouth. The implant analogs were precisely inserted into the impression's appropriate mounts. In the lab, self-curing acrylic resin was used to build the implant verification jig (IVJ). After the material set was divided into four portions by disc and numbered on a working model, as shown in Figure 3(a), 3(b), and 3(c), the long transfer copings were splinted on the cast. An open-tray approach was used to capture the final impression.

For a full denture, it is necessary to capture the ridge, and all anatomical areas, supra-implant monoprint (DETAX Germany) was injected under and around the jig. As shown in Figure 3(d) and 3(e), the custom tray was positioned so that the guide pinheads were visible through the tray. To prevent having to remake the framework, the final verification jig was created using stiff poly (methyl methacrylate) (PMMA) materials using CAD–CAM technology and tested on the patient. All patients' jaw relationships were recorded using the same fundamental guidelines. In the try-in stage, the vertical dimension of occlusion, COR, esthetics, and occlusion were assessed in the patient's mouth. The tooth arrangement was the basis for the framework's and bar's design.

#### 2.3.1. CAD/CAM Fabrications of the BioHPP Framework and Bar

**Figure 4:**
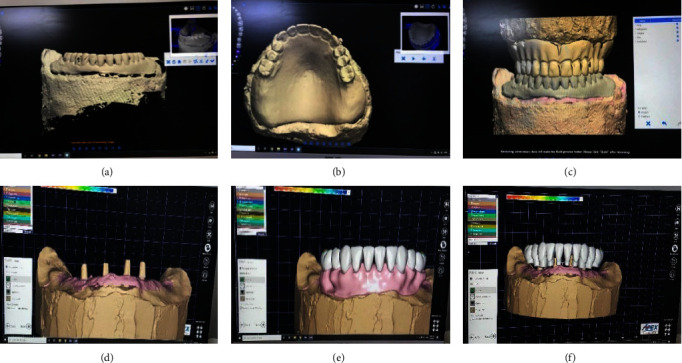
STL files of scanned; (a) the wax-up lower denture, (b) the antagonist denture, (c) mounting upper and lower casts, (d) abutments after spray application abutments, (e) generated STL files were imported, and (f) the files overlapped each other.

**Figure 5:**
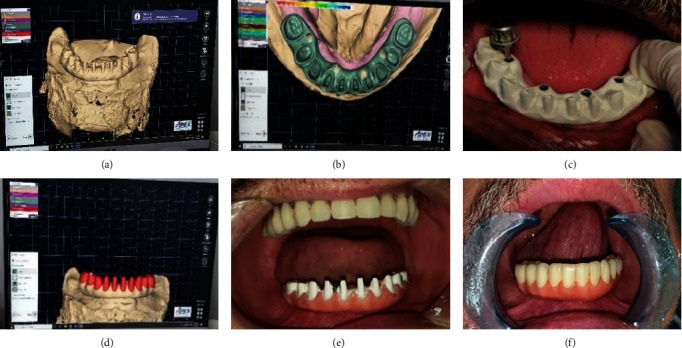
(a, b) The virtual cutback is performed in a screw-retained framework with individual abutment preparations for future crown cementations, (c) framework trying to be patient, (d) completed BioHPP framework, and (e, f) final superstructure delivery.

The try-in received a spray for digital scanning. With the help of a lab scanner, the lower wax-up denture, the opposing denture, and the semiadjustable articulator with the upper and lower casts mounted on it were all individually scanned, as shown in Figure 4(a)–4(f). The STL files were then created, and the main window of the Exocad program (Exocad Dental CAD 2016 GmbH, Darmstadt, Germany) was launched for Group 1 (the BioHPP hybrid prosthesis). It was crucial to choose the following steps: reduced wax-up, adjacent teeth, antagonist teeth, and pontic wax-up; next, the type of restoration and material that was designed; and finally, just the implants were imported into a CAD program (Exocad Dental CAD 2016 GmbH, Darmstadt, Germany), and the files were layered on top of one another. A virtual cutback was carried out using CAD software to create a framework with individual abutment preparations for expected multiple crown cementation that is screw retained. The 10 different abutment preparations used in the CAD/CAM-milled BioHPP framework were tested, and the fit was verified clinically and radiographically. The final PMMA resin crowns (Dental VIPI Ltd.; VIPI Block Trilux) were designed and manufactured digitally using the BioHPP framework, which was scanned in the lab and stored as STL files in the CAD software, as illustrated in Figure 5(a)–5(f).

**Figure 6:**
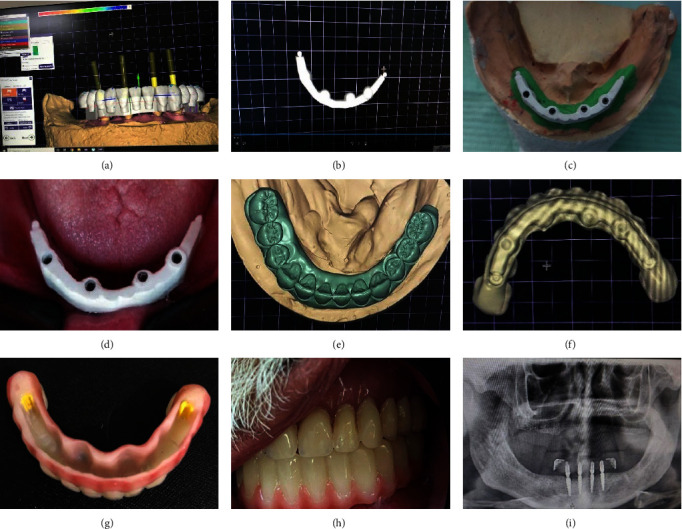
Illustration of the BioHPP bar construction using CAD–CAM (a, b), the BioHPP bar on the cast, intraoral (c, d), superstructure designed in PMMA (e, f), final prosthesis fitting surface (g), intraoral view after delivery (h), and postoperative panorama radiographic (i).

Similar steps for prostheses were mentioned. Group II (BioHPP bar overdenture). The Dolder bar was selected and designed from the present bar profile (12. VSS Vario Soft Bar (VSP-F)) with two vertical stud attachments on a cantilever with a minimum extension and a posterior parallel-walled segment (Variosoft VS3-Mini Attachments, Bredent, Germany) were selected from the present library in the Exocad software. The tissue bar is sized to provide a 1-2 mm clearance to aid with oral hygiene, with the bar having a 5 mm height and a 3.5 mm width. Once the plan was complete, the CAM machined the PMMA verification jig, and to verify passive fitting, it was tested within the patient's mouth. The BioHPP blank was clamped to the milling fixture and milled on an exact 5-axis milling unit as shown in Figure 6(a)–6(i).

### 2.4. Evaluation of Patients

#### 2.4.1. Radiographic Evaluation

**Figure 7:**
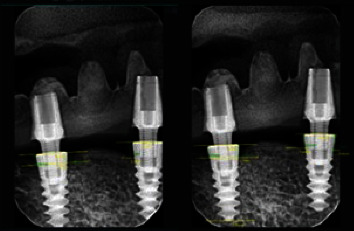
Linear measurement of mesial and distal MBL.

The GXS-700-DIGITAL intraoral sensor (GENDEX-USA) and a specially built acrylic template were used to take intraoral radiographs with the standardized long cone paralleling technique to measure changes in the peri-implant crestal bone level. Follow-up appointments were planned for denture insertion. Periods of 6, 12, and 18 months after the superstructures were put in (as shown in Figure 7). To check the prosthesis and gather information (radiographic evaluation).

#### 2.4.2. Patient Satisfaction

The patient's satisfaction was measured using a VAS questionnaire. Questionnaires evaluating patient satisfaction with rehabilitation were distributed to the patients. At each follow-up appointment, the questionnaires were given to the patients before denture insertion and after implant rehabilitation [26] at 6 months (T6 m) and 18 months (T18 m) after the insertion of the superstructure. On a scale of 1–5, six variables were evaluated: speaking, chewing, comfort, cosmetic oral hygiene, and overall satisfaction (very satisfied = 5, satisfied = 4, fair = 3, dissatisfied = 2, and highly unhappy = 1).

## 3. Results

The mean and standard deviation of all data were displayed. Three tables were used to display the data. SPSS 16® (Statistical Package for Scientific Studies), GraphPad Prism, and Windows Excel were used for the statistical analysis.

The Shapiro–Wilk test and Kolmogorov–Smirnov test for normality were used to extract the quantitative data, and the results showed that the alternative hypothesis was rejected since the significant level (*p* value) was negligible and *p* value >0.05. The results were drawn using parametric data with a normal distribution that resembled a normal bell curve. In light of this, an independent *t*-test was used to compare the two groups. However, numerous comparisons were performed to compare distinct periods using the repeated one-way ANOVA test and Tukey's post hoc test. An alternative method used the *χ*^2^ test to compare qualitative data (patient satisfaction).

### 3.1. Amount of Bone Loss

Repetitive one-way ANOVA revealed a significant difference between the two groups in the amount of bone loss in the mesial and distal surfaces over time. Tukey's post hoc test for multiple comparisons was then used to determine whether the difference was statistically significant.

The overall MBL around the surfaces of the posterior implant from 0 (insertion) to 18 months were (0.0.33 ± 0.0125) and (0.22 ± 0.0085 mm) for Group I (BioHPP hybrid prosthesis) and Group II (BioHPP bar overdenture), respectively while from 0 to 12 months, the total MBL for all of implants surfaces was 0.245 ± 0.01 and 0.18 ± 0.007 mm for Groups I and II, respectively. From insertion to 6 months, the complete change in alveolar bone height for all surfaces of the implants was 0.13 ± 0.0065 and 0.1 ± 0.0045 mm for Groups I and II, respectively.

The overall MBL around the surfaces of the anterior implant from 0 (insertion) to 18 months was 0.39 ± 0.015 and 0.26 ± 0.011 mm for Group I (BioHPP hybrid prosthesis) and Group II (BioHPP bar overdenture), respectively, while from 0 to 12 months, the overall crestal bone loss for all surfaces of the implants were 0.315 ± 0.0125 and 0.185 *a* ± 0.009 mm for Groups I and II, respectively. From insertion to 6 months, the time intervals in alveolar bone height change for all surfaces of implants were (0.185 ± 0.009) and (0.155 ± 0.0075 mm) for Groups I and II, respectively. A significantly greater bone loss was detected for Group I (BioHPP hybrid prosthesis) than for Group II (BioHPP bar overdenture). Additionally, a comparison between Groups I (BioHPP hybrid prosthesis) and II (BioHPP bar overdenture) was made using an independent *t*-test, and the results are shown in Table 1. Group I was significantly more than Group II at all intervals in mesial and distal surfaces and overall anterior and posterior implants (*P* < 0.05).

### 3.2. Patient Satisfaction

The patient satisfaction survey results showed that, after 6 months, there was an insignificant difference between them all (*P* > 0.05) except for the comfort for the overdenture group, (3.14 ± 0.38), while the fixed hybrid was 4.00 ± 0.00. After 18 months, the comparison between both groups revealed an insignificant difference (*P* > 0.05) in speech, chewing, esthetics, oral hygiene, and general satisfaction. While patient comfort was 4.43 ± 0.53 and 5.00 ± 0.00, for Group II (BioHPP bar overdenture) and Group I (BioHPP hybrid prosthesis), respectively, revealed there was a significant difference as presented in (Tables 2 and 3): illustration of patients' satisfaction between Group I (BioHPP hybrid prosthesis) and Group II (BioHPP bar overdenture).

## 4. Discussion

Due to poor stability, retention, and chewing capacity, individuals with edentulous mouths typically face problems with their entire mandibular dentures. The insertion of implants creates a more favorable restoration in such patients [27]. To restore function and esthetics, enhance masticatory effectiveness, and enhance patient satisfaction, oral rehabilitation with implant-supported prostheses for an edentulous arch using implant-supported fixed and detachable prostheses is a common treatment option. In contrast, overdenture mechanical retention issues, screw loosening or fracture, and implant fractures were mechanical difficulties. Additionally, phonological and esthetic problems were shown in several studies [2].

Patients were carefully chosen per predetermined criteria to minimize human variables and avoid adverse variables influencing the study's outcomes. A minimum of 15 mm interarch space has been recommended for fixed prostheses supported by mandibular implants or bar overdentures [28]. The patients with adequate buccolingual width at implant placement sites were involved to ensure that 1 ml of buccal and lingual bone thickness remained on the implant after its installation to enhance osseointegration [29]. The current study used computerized treatment planning and surgical guide to achieve implant location and alignment consistency and lower operator variability [30].

In the current study, implant location, and alignment standardization were achieved using computer-generated treatment planning and surgical guide design, reducing operator variability [31, 32]. Therefore, with the dual-scan technique, the patient's existing prosthesis is used by biting on cotton rolls bilaterally in centric occlusion to adapt the dentures accurately and serves as a radiological guide to visualize the mandibular anatomy and architecture [32]. The length and width of the implants were standardized in all cases. The implants used were two piece, threaded, self-tapping, and root-form implants measuring 10 mm long and 4.2 mm in width. This implant design improves the contact area between the implant and the surrounding bone for better osseointegration and to ensure primary stability throughout the early healing period [33].

All patients had complete dentures made using standard clinical and laboratory protocols. Also, the same materials were used to remove any factors that could affect the results of this study. Several materials and their combinations have been used to manufacture frameworks; high frequency of veneer chipping is a common prosthodontic complication of restorations with a titanium framework. BioHPP has recently attracted more attention as a dental material for creating superstructures on dental implants. It is based on a PEEK polymer [34]. Many clinical studies show that BioHPP could be used as an alternative framework material to support complete-arch restorations [24, 35, 36].

The CAD/CAM framework and bar have proven to be more accurate, less time-consuming, and less expensive. These findings improve treatment time, patient experience, and accessibility [37, 38]. Three items were required for scanning (the lower wax-up denture, the opposing arch, and the mounting costs on the articulator). This technique makes the traditional fabrication of silicone keys unnecessary since the STL files contain all the necessary information for tooth position, contours, and spatial orientation [39, 40].

The milled-bar architecture had a parallel design and included retention devices used in the posterior bar extensions (Bredent, Germany) that snap into the fitting surface of the PMMA overdenture, providing excellent stability, and additional retention, often without a flange. A fixed-removable implant solution that offers a design optimal for implant placement [41]. The peri-implant marginal bone level was assessed using digital periapical radiographs and the paralleling technique that provided an image with minimal distortion and can thus be used for determining the vertical and mesiodistal dimensions of the edentulous mandibular area being examined [42, 43].

The appropriate case selection, implant placement, angulation, and opposing occlusion to complete dentures are all aspects that ensured the crestal bone height decrease in both groups was within an appropriate range and that completed rehabilitation with BioHPP materials [44]. Also, BioHPP rehabilitation considerably reduces peak masticatory forces for both vertical and lateral movement compared to titanium, zirconium, or ceramic. This characteristic positively influences the patient and increases the restoration's durability, particularly for large stretched frames [45].

CAD/CAM fabrication of bars and frameworks has eliminated distortion, gives a better fit, and has fewer fabrication steps than conventional casting techniques. Implant-supported fixed prosthesis with CAD/CAM milled framework is reliable [46]. Research has demonstrated that main structures built using CAD/CAM procedures offer greater passive fit and minor volumetric misfits than casting processes. Clinical studies with a follow-up period of 10 years showed fewer technical complications of the CAD/CAM structures than casting structures [47–51].

The success of implant treatment is based on the health of the peri-implant soft and hard tissues. To be successful, the implant must show no pain, inflammation, or infection. For the success of the implant, it had been thought in the early 1990s that a minimal bone loss of 1.0–1.5 mm in the first year, followed by an annual bone loss of 0.1–0.2 mm, was acceptable. This study found that MBL is lower in patients who receive prosthetic restorations with a BioHPP bar implant-supported and retained overdenture (Group II) than in patients who receive a BioHPP hybrid prosthesis (Group I) during the first 6 months after loading. It may be explained by the resilient nature of the connection between the overdenture and the attachment used in this study (a bar attachment), i.e., it allows some movement. Less bending strain was developed in the mandible with overdentures. The study shows that distal extensions included in bars affected neither the degree of distal bone loss nor the implant survival rate [52].

Reduced bone resorption of the milled bar may result from effective splinting of the implants by the hard-milled bar. This study agreed with Pozzi et al. [53], and they used a CAD/CAM titanium milled bar to assess bone resorption at four implant-supported overdentures. They discovered a mean bone resorption of 0.29 ± 0.16 mm at the 1-year follow-up.

Regarding patient satisfaction, significance was only found in the pain/discomfort and hygiene maintenance categories in this study's first follow-up (6 months) using the VAS. Patients more easily clean overdentures than fixed prostheses. This outcome is also not unexpected, considering that a systematic review concluded that five clinical studies all agreed that it is more difficult for patients to maintain oral hygiene with implant-supported fixed prostheses [54]. The most common complication after delivering an implant-supported hybrid prosthesis has been mucositis due to improper oral hygiene [55].

All patients reported few postinsertion issues over the 18-month follow-up period, and all implants used and examined in the study had good results within the parameters studied. The equal satisfaction between the two treatment options may be due to using the same number of implants and superstructure materials. It might be connected to the beneficial BioHPP-reinforced polymer: restorations with low specific weight, bone-like flexibility, shock absorption, minimal material fatigue, absence of viscoplastic fractures, excellent biocompatibility, minimal plaque buildup, and lack of corrosion or color stability [56, 57].

PEEK has been used in many trials to aid in retaining overdentures supported by implants. PEEK provided more retention than conventional materials, according to Sharaf et al. [58], and this resulted in higher patient satisfaction. Another trial [59] evaluated clinical, prosthetic, and patient outcomes of a milled bar with PEEK and metal housings for inclined implants supporting mandibular overdentures. After a year-long follow-up, the metal group showed a significantly higher plaque score and marginal bone resorption than the PEEK group. Additionally, the PEEK group reported higher satisfaction with retention, stability, speech, and esthetics. Additional research comparing the BioHPP fixed detachable prosthesis and BioHPP bar with a more significant number of patients and a more extended follow-up period (to examine the effect of time on patient satisfaction) to confirm the long-term validity of the results of this study is advised. One limitation of this research was the limited follow-up period of 18 months to examine the effect of time on the MBL around implants and patient satisfaction.

## 5. Conclusion

BioHPP framework material is an alternative material for implant rehabilitation of the edentulous mandible. A minimal MBL was noticed in overdentures compared to hybrid prostheses when using BioHPP. Regardless of the prosthetic design, high patient satisfaction was found.

## Figures and Tables

**Table 1:** Comparison between Group I (BioHPP hybrid prosthesis) and Group II (BioHPP bar overdenture) regarding the amount of bone loss of mesial and distal surfaces of anterior and posterior implants at different intervals.

			0–6 months		0–12 months		0–18 months		*P* value
			*M*	SD	*M*	SD	*M*	SD	
Posterior	Distal	Group I	0.14^a^	0.007	0.26^b^	0.011	0.35^c^	0.013	<0.0001^*∗*^
		Group II	0.09^a^	0.004	0.16^b^	0.006	0.21^c^	0.008	<0.0001^*∗*^
		*P* value	<0.0001^*∗*^		<0.0001^*∗*^		<0.0001^*∗*^		
	Mesial	Group I	0.12^a^	0.006	0.23^b^	0.009	0.31^c^	0.012	<0.0001^*∗*^
		Group II	0.11^a^	0.005	0.2^b^	0.008	0.23^c^	0.009	<0.0001^*∗*^
		*P* value	<0.0001^*∗*^		<0.0001^*∗*^		<0.0001^*∗*^		
	Overall	Group I	0.13	0.0065	0.245	0.01	0.33	0.0125	<0.0001^*∗*^
		Group II	0.1	0.0045	0.18	0.007	0.22	0.0085	<0.0001^*∗*^
		*P* value	<0.0001^*∗*^		<0.0001^*∗*^		<0.0001^*∗*^		

Anterior	Distal	Group I	0.14^a^	0.007	0.22^b^	0.009	0.28^c^	0.011	<0.0001^*∗*^
		Group II	0.13^a^	0.006	0.19^b^	0.008	0.21^c^	0.009	<0.0001^*∗*^
		*P* value	0.004^*∗*^		<0.0001^*∗*^		<0.0001^*∗*^		
	Mesial	Group I	0.23^a^	0.011	0.41^b^	0.016	0.5^c^	0.019	<0.0001^*∗*^
		Group II	0.18^a^	0.009	0.27^b^	0.011	0.31^c^	0.013	<0.0001^*∗*^
		*P* value	<0.0001^*∗*^		<0.0001^*∗*^		<0.0001^*∗*^		
	Overall	Group I	0.185^a^	0.009	0.315^b^	0.0125	0.39^c^	0.015	<0.0001^*∗*^
		Group II	0.155^a^	0.0075	0.23^b^	0.0095	0.26^c^	0.011	<0.0001^*∗*^
		*P* value	<0.0001^*∗*^		<0.0001^*∗*^		<0.0001^*∗*^		

M, mean; SD, standard deviation.  ^*∗*^Significant difference (*P* < 0.05). ^a,b,c^Values with the same superscript letters were insignificantly different (*P* > 0.05) whereas values with different superscript letters were significantly different (*P* ≤ 0.05).

**Table 2:** The mean, standard deviation (SD) values, and results of independent *t*-test for comparison between patient satisfaction scores in the two groups at 6 months.

6 months	Group 1	Group 2	Test value	*P* value	Sig.
Speech	4.14 ± 0.38	4.00 ± 0.00	1.000	0.337	NS
Chewing	4.14 ± 0.38	4.29 ± 0.49	−0.612	0.552	NS
Comfort	3.14 ± 0.38	4.00 ± 0.00	−6.000	0.000	HS
Aesthetic	4.57 ± 0.53	4.43 ± 0.53	0.500	0.626	NS
Oral hygiene	3.14 ± 0.38	3.43 ± 0.53	−1.155	0.271	NS
General satisfaction	3.83 ± 0.21	4.03 ± 0.21	−1.750	0.106	NS

**Table 3:** The mean, standard deviation (SD) values, and results of independent *t*-test for comparison between patient satisfaction scores in the two groups at 18 months.

18 months	Group 1	Group 2	Test value	*P* value	Sig.
Speech	4.71 ± 0.49	4.86 ± 0.38	−0.612	0.552	NS
Chewing	4.86 ± 0.38	4.71 ± 0.49	−0.612	0.552	NS
Comfort	4.43 ± 0.53	5.00 ± 0.00	−2.828	0.015	S
Aesthetic	5.00 ± 0.00	4.71 ± 0.49	1.549	0.147	NS
Oral hygiene	4.00 ± 0.58	4.43 ± 0.79	−1.162	0.268	NS
General satisfaction	4.60 ± 0.16	4.74 ± 0.25	−1.263	0.23	NS

## Data Availability

The research ethics committee reviewed this clinical trial (eth no. 686) of the Faculty of Dentistry at Ain Shams University, ethicscommittee.fdasurec@gmail.com and ClinicalTrials.gov.
